# *Acanoides* gen. n., a new spider genus from China with a note on the taxonomic status of *Acanthoneta* Eskov & Marusik, 1992 (Araneae, Linyphiidae, Micronetinae)

**DOI:** 10.3897/zookeys.375.6116

**Published:** 2014-01-30

**Authors:** Ning Sun, Yuri M. Marusik, Lihong Tu

**Affiliations:** 1 College of Life Sciences, Capital Normal University, Xisanhuanbeilu Str. 105, Haidian Dist., Beijing, 100048, P. R. China; 2 Institute for Biological Problems of the North, FEB Russian Academy of Sciences, Portovaya Str. 18, Magadan, 685000, Russia; 3 Zoological Museum, University of Turku, FI-20014 Turku, Finland

**Keywords:** Taxonomy, new species, new genus, genitalic morphology, movable epigynum

## Abstract

A new “micronetine” genus *Acanoides* **gen. n.** is erected to accommodate two species from China: *Acanoides beijingensis* **sp. n.** as the type species and *Acanoides hengshanensis* (Chen & Yin, 2000), **comb. n.**, with the females described for the first time. The genitalic characters and somatic features of the new genus were studied by means of light microscopy and scanning electron microscopy (SEM). The monophyly of the new genus was tested by a phylogenetic analysis based on molecular data. Descriptions of the new genus, the new species and the new combination are presented; SEM images and microscopy pictures of somatic and genitalic characters are provided in detail. To distinguish from other genera with similar genitalic characters, we compare the new genus with the species of *Acanthoneta* Eskov & Marusik, 1992, *Epibellowia* Tanasevitch, 1996 and *Wubanoides* Eskov, 1986. Four putative synapomorphies for *Acanoides* **gen. n.** are suggested to support its monophyly that could be tested in the future. Furthermore, redescriptions of the epigynal morphology of *Acanthoneta aggressa* Chamberlin & Ivie, 1943 (Nearctic) and on the male of *A. dokutchaevi* Eskov & Marusik, 1993 (Far East Asia, firstly recorded from China) are provided. Based on comparison with *Poeciloneta*, from which *Acanthoneta* **stat. n.** was separated by Saaristo and Tanasevitch (1996), a revised diagnosis is proposed to support the generic status.

## Introduction

Micronetinae Hull, 1920 is a fairly large subfamily of Linyphiidae Blackwall, 1859, including 1199 species placed in 90 genera (Tanasevitch 2014). It was redelimited by Saaristo and Tanasevitch (1996), who established eight new genera for 89 species, and raised three subgenera to generic status. Subsequently, a series of revisions were published (e.g. Saaristo and Tanasevitch 2002a, 2002b; Saaristo and Marusik 2004; Saaristo et al. 2006; Tu et al. 2006; Tu and Li 2006) that resulted in many new genera and a new subfamily Ipainae Saaristo, 2007. Results of these series of review works have not been tested in a phylogenetic context; neither Micronetinae nor Ipainae monophyly, as well as that of the genera included in the two subfamilies have been tested so far.

*Poeciloneta hengshanensis* (Chen & Yin, 2000) from China, originally placed in *Lepthyphantes* Menge, 1866, has its male palp very similar to that of *Poeciloneta (Acanthoneta) aggressa* (Chamberlin & Ivie, 1943). *Acanthoneta* Eskov & Marusik, 1992 is one of the three genera raised from subgeneric status by Saaristo and Tanasevitch (1996) with the type species *Acanthoneta aggressa*. Tu et al. (2006) transferred *Poeciloneta hengshanensis* to *Acanthoneta* based on the similarity of the male palpal morphology. However, raising *Acanthoneta* to a generic status “was not accompanied by a diagnosis or justification”, and hence not accepted in The World Spider Catalog (Platnick 2014). All members of *Acanthoneta* are currently placed in *Poeciloneta* Kulczyński, 1894.

Females of *Poeciloneta hengshanensis* (previously unknown) were found in new material from China. However, its epigynal conformation is neither congruent with that of *Poeciloneta aggressa*, nor with any other species of *Poeciloneta*. Based on the presence of an extensible basal part, the movable epigynum accords with the diagnosis of the subfamily Ipainae Saaristo, 2007 (for example *Ipa* Saaristo, 2007 and *Solenysa* Simon, 1894). Additionally, we found another new species with genitalic morphology very similar to that of *Poeciloneta hengshanensis*: the male palpal morphology similar to *Acanthoneta* and a movable epigynum in accordance with ipaine type.

A new genus *Acanoides* gen. n. is erected here for these two species. To test the placement of the new genus within Linyphiidae, a phylogenetic analysis based on newly sequenced molecular data of the two species and that of other linyphiids downloaded from NCBI was conducted. In the present study, the two species and the new genus are described. Characters of copulatory organs and somatic features of both species are illustrated by means of SEM and light microscopy. To distinguish the new genus from other “micronetine” genera with similar male palpal morphology and ipaine genera with a similar movable epigynum, the new genus is compared with the genera *Acanthoneta* (Micronetinae), *Wubanoides* Eskov, 1986 and *Epibellowia* Tanasevitch, 1996 (Ipainae). Due to limited material available for examination, comparisons are largely based on descriptions and illustrations provided by Tanasevitch (1996), Saaristo and Tanasevitch (2000) and Saaristo (2007); images of the epigynum of *Acanthoneta aggressa* and the male of *Acanthoneta dokutchaevi* Eskov and Marusik, 1994 are presented here. Four putative synapomorphies are suggested for *Acanoides* gen. n. that could be tested in future study. In addition, diagnoses for *Acanthoneta* stat. n. are provided based on comparison with illustrations of genitalic characters provided by Saaristo and Tanasevitch (2000), to support its generic status proposed by Saaristo and Tanasevitch (1996). The composition and monophyly of both *Acanoides* gen. n. and *Acanthoneta* stat. n. could be tested in future study.

## Materials and methods

Specimens were examined and measured using a Leica M205A stereomicroscope. Male palps and epigyna were examined after they were dissected from the body. Left structures (e.g. palps, legs, etc.) were depicted. Embolic divisions were excised by breaking the membranous column which connects the suprategulum and radix. Male palps and epigyna were cleared in methyl salicylate. Digital images were taken with a Leica DFC 500 camera, as composites of multiple focus images assembled using the software package LEICA APPLICATION SUITE. Scanning electron microscopy (SEM) images were taken using a S-3400N scanning electron microscope at the China Agricultural University. For SEM examination the specimens were prepared following Álvarez-Padilla and Hormiga (2008). SEM images of the embolic division taken from the right palp were mirrored to match those taken from the left palp. All measurements were taken with a micrometer and are expressed in millimeters. The leg measurements are given in the following sequence: total (femur, patella+tibia, metatarsus, tarsus). All specimens examined here are deposited in the College of Life Sciences, Capital Normal University, China (CNU) and in the College of Life Sciences, Hunan Normal University, China (HNU), except for the female material of *Acanthoneta aggressa*, the epigynal pictures of which were provided by Don Buckle (Saskatoon, Canada). Distribution data for these species within China are presented at the provincial level. Terminology for the epigynal characters follows Tu and Hormiga (2010) and male palpal and somatic characters follows that of Saaristo and Tanasevitch (1996) and Hormiga (2000). Anatomical abbreviations used in the text and figures:

### Somatic morphology

AER anterior eye row

ALE anterior lateral eye(s)

AME anterior median eye(s)

AMEd diameter of AME

PER posterior eye row

PLE posterior lateral eye(s)

### Male palp

AX apex of embolus

DM distal membrane of terminal apophysis

DSA distal suprategular apophysis

EM embolic membrane

EP embolus proper

FiG Fickert’s gland

LC lamella characteristica

P paracymbium

PCA proximal cymbial apophysis

PH pit hook

R radix

SE serrated area on embolus

SPT suprategulum

TA terminal apophysis

TH thumb of embolus

### Epigynum

CO copulatory opening

CG copulatory groove

DP dorsal plate

EA extensible area of epigynal basal part

EB epigynal basal part

FG fertilization groove

MP median plate

S spermathecae

SC scape

ST stretcher

VP ventral plate

### Phylogenetic analysis

Based on the dataset of Arnedo et al. (2009) which includes 34 linyphiid taxa (*Erigone dentipalpis* was not included as it has only one of the five genes available), newly sequenced data of the two *Acanoides* and data of another 65 linyphiid taxa downloaded from NCBI were added. A total of 111 taxa were sampled in our matrix, ten outgroup taxa of other araneoid families as in that of Arnedo et al. (2009) and 101 ingroup taxa, which cover the representatives of all the seven subfamilies currently proposed; one *Solenysa*, as a representative of ipaine, and *Acanthoneta* were included to test the placement of *Acanoides*.

Five genes: cytochrome c oxidase subunit I (CO1) and 16S rRNA (16S) and three nuclear genes 18S rRNA (18S), 28S rRNA (28S) and Histone H3 (H3) were sequenced for *Acanoides beijingensis* sp. n. and *Acanoides hengshanensis*. Molecular procedures for sequencing follow that of Arnedo et al. (2009), with the same molecular markers to maximize the overlap of dataset. Taxa sampled and sequence accession numbers are presented in Appendix - Table S1. Data were automatic multiple aligned using the computer program Clustal X version 1.81 (Thompson et al. 1997). Gaps were treated as missing data. Maximum Likelihood analysis was performed using RAxML v7.2.7 as implemented on the Cipres Gateway (Miller et al. 2010). Bootstrap support analysis was performed with the commands: raxmlHPC-HYBRID-7.3.1 -T 6 -s infile -n result -x 12345 -p 876 -f a -N 1000 -m GTRCAT -q part.

## Results

**Figure S1.**
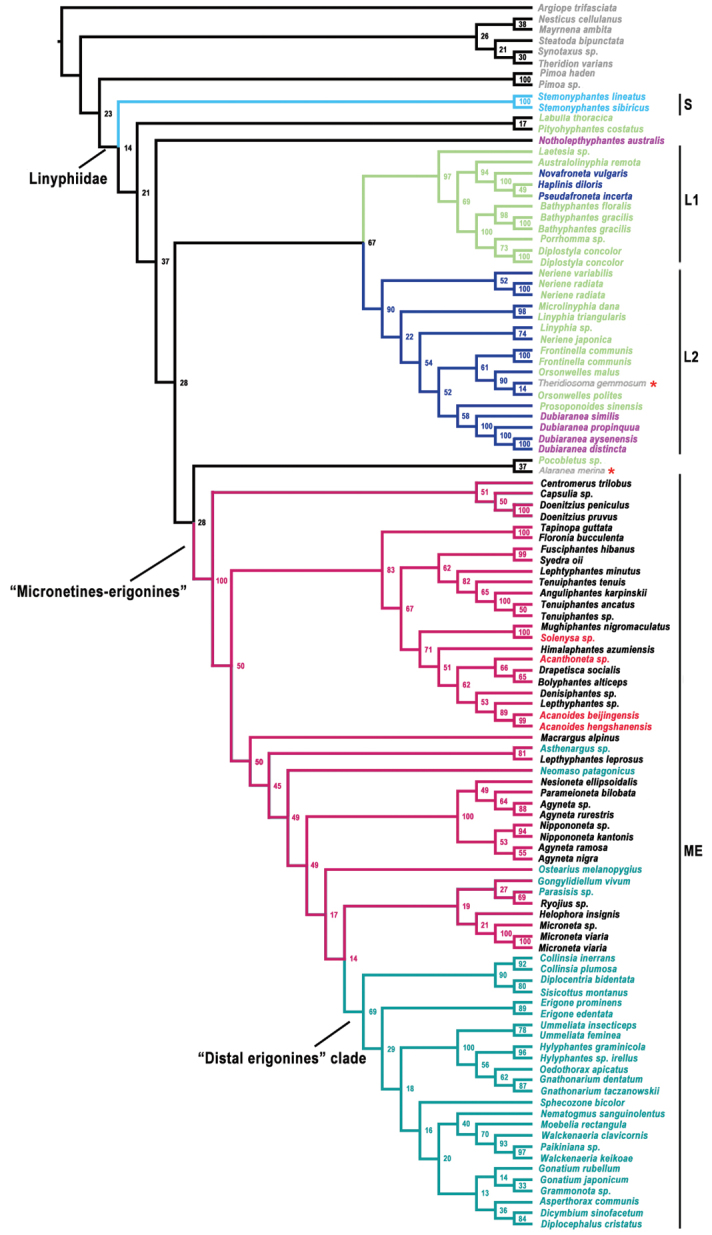
Linyphiid phylogeny resulting from Maximum Likelihood analysis based on molecular data. Numbers at the nodes are bootstrap value. Branches in color indicate the four robustly supported clades within linyphiids: **S** *Stemonyphantes* clade (blue) **L1** “linyphiines”-1 clade (pale green) **L2** “linyphiines-2” clade (dark blue) **ME** “micronetines-erigonines” clade (red, with “Distal erigonines” clade in green). Taxa in different colors sampled from different groups: grey, outgroup; blue, Stemonyphantinae; pale green, Linyphiinae; dark blue, Mynogleninae; pink, Dubiaraneinae; black, Micronetinae; red, Ipainae, *Acanthoneta* and *Acanoides* gen. n.; green, Erigoninae. Red stars indicate the two out-group taxa: cyatholipid *Alaranea* and theridiosomatid *Theridiosoma* embedded within Linyphiidae.

All five genes were sequenced for *Acanoides beijingensis* sp. n. and *Acanoides hengshanensis* (Appendix - Table S1). The monophyly of Linyphiidae and its sister relationship with Pimoidae were not recovered in the result of phylogenetic analysis as two outgroup taxa: cyatholipid *Alaranea* and theridiosomatid *Theridiosoma* are embedded within Linyphiidae (Appendix - Fig. S1). Besides some weakly supported deeper branches, four robustly supported clades are recognized: *Stemonyphantes* clade (clade S), “micronetines-erigonines” clade (clade ME), “linyphiines”-1 clade (clade L1) and “linyphiines”-2 (clade L2). For the seven subfamilies currently proposed, only Stemonyphantinae Wunderlich, 1986 (the *Stemonyphantes* clade) and Mynogleninae Lehtinen, 1967 are monophyletic, while the mynoglenines clade and the *Dubiaranea* clade fall into clades L1 and L2 respectively that make Linyphiinae Blackwall, 1859 become a paraphyletic group; taxa of Micronetinae form a paraphyletic group, nested with taxa of Ipainae and Erigoninae within clade ME. The two *Acanoides* species form a robustly supported monophyly, distantly related to *Acanthoneta* and *Solenysa*.

## Discussion

The result of the phylogenetic analysis based on molecular data suggests that the new species from Beijing is the sister taxon of *Poeciloneta hengshanensis* which had ever been transferred to *Acanthoneta* by Tu et al. (2006). The lineage comprised by the two species is distantly related to *Acanthoneta* sp. (Appendix - Fig. S1). Accordingly, we erected here *Acanoides* gen. n. to accommodate the two species: *Acanoides beijingensis* sp. n. and *Acanoides hengshanensis* comb. n. The three known *Acanthoneta* species have very distinct male palpal morphology, only differ from that of the type species in small details (Eskov and Marusik 1992, 1993). Regardless the *Acanthoneta* taxon is congeneric with, or is the type species *Acanthoneta aggresus*, the new genus differs from all the three known species of *Acanthoneta* as well as all other “micronetines” in the females having a movable epigynum (Figs 4G, 5G) and the males having a longer and sharper embolus proper (Figs 2D, 3D) which generally is pointed in “micronetines” (Fig. 6F); Fickert’s gland located in the membranous area outside the radix (Figs 2D, 3D), rather than embedded within the radix as usually the case in “micronetines” (Fig. 6G). This suggests that the two species are not congeneric with *Acanthoneta*.

Our results suggest an unknown *Lepthyphantes* species as a sister group to the *Acanoides* clade. *Lepthyphantes* Menge, 1866, which includes almost 500 species, is not a natural group (Saaristo and Tanasevitch 1996). All *Lepthyphantes* species, except five, have been transferred or are waiting to be transferred to other genera (e.g. Saaristo and Tanasevitch 1996, 2002a, b; Saaristo and Marusik 2004; Tu et al. 2006). The position of *Lepthyphantes* sp. on the tree indicates it is neither *Acanthoneta*, nor *Lepthyphantes*. Nevertheless, without morphological data, we fail to determine whether *Lepthyphantes* sp. is as a sister group of, or a number of *Acanoides* gen. n., so the close relative of *Acanoides* remains unresolved.

The genitalic characters of *Acanoides* make its subfamily placement problematic due to the epigynal character in accordance with Ipainae type, but the male palpal morphology of the “micronetine” type. Redelimitation of Mironetinae (Saaristo and Tanasevitch 1996) and the series of revisions of “micronetine” genera (e.g. Saaristo and Tanasevitch 2002a, 2002b; Saaristo and Marusik 2004; Saaristo et al. 2006; Tu et al. 2006; Tu and Li 2006) resulted in many new genera and even a new subfamily Ipainae (Saaristo 2007). However, none of them has been tested in a phylogenetic framework. Results of the first phylogenetic analysis for linyphiids based on molecular data indicate that neither Micronetinae nor Ipainae is a monophyletic group (Arnedo et al. 2009). Such a result was recovered in the present study too: “micronetine” taxa formed a paraphyletic group, and movable epigynum independently evolved in *Acanoides* and *Solenysa* (Appendix - Fig. S1). The extensible solenoid serving as a synapomorphy for *Solenysa* (Tu & Hormiga, 2011), the ventrally folded extensible epigynal basal part, together with long and sharp embolus proper, slender and unbranched lamella characteristica, and outside radix located Fickert’s gland are four putative synapomorphies for *Acanoides* gen. n.

With greatly increased ingroup sampling, the result of the present study produce a similar topology with that of Arnedo et al. (2009): four strongly supported clades S, L1, L2 and ME that correspond to the*Stemonyphantes* clade, clades C and D, and the “micronetines-erigonines” clade in the latter (Appendix - Fig. S1). Most newly added taxa fell into the clade ME that enriched the topology. However, the problems left by the previous study (Arnedo et al. 2009), such as the monophyly of Linyphiidae, placements of the weakly supported deeper branches, and taxa of different subfamilies placed together rendering most of the traditionally recognized subfamilies non monophyletic, persist. Six of the seven subfamilies currently proposed are not monophyletic groups. The higher level relationships within linyphiids reflected by phylogenetic result are still far away from the classic subfamily system (see Millidge 1984, 1993; Saaristo and Tanasevitch 1996; Saaristo 2007). Nevertheless, revising the whole higher level linyphiid systematics is beyond the scope of the present study. In the text bellow we keep using Micronetinae and Ipainae following the current taxonomic system.

Although with ingroup sampling about two times increased, the sampling size of the current dataset seems not to be enough to resolve the placements of *Acanoides* and *Acanthoneta*, as well as *Poeciloneta*, from which *Acanthoneta* were separated (Eskov and Marusik 1992), their close relatives, and the relationships among them. To better understand the higher level phylogenetic relationships of linyphiid spiders, more information, such as morphology and behavior, and a comprehensive sampling design are necessary. Here, we provide four putative synapomorphies for the new genus *Acanoides* that could be tested in future phylogenetic studies.

## Taxonomy

### Linyphiidae Blackwall, 1859

#### Acanoides gen. n.

http://zoobank.org/4632240B-5228-4EB7-A1BC-CBD9176FEC2B

http://species-id.net/wiki/Acanoides

##### Type species.

*Acanoides beijingensis* sp. n.

##### Composition.

Two species, *Acanoides beijingensis* sp. n. and *Acanoides hengshanensis* (Chen & Yin, 2000) comb. n.

##### Diagnosis.

The males of *Acanoides* gen. n. can be distinct from *Acanthoneta* by the sharp embolus proper, the slender lamella characteristica unbranched, and by the Fickert’s gland located in the membranous area outside the radix (Figs 2D, 3D). The females can be distinguished by the ventrally folded extensible epigynal basal part (Figs 2F, 3F).

##### Description.

**Figure 1.**
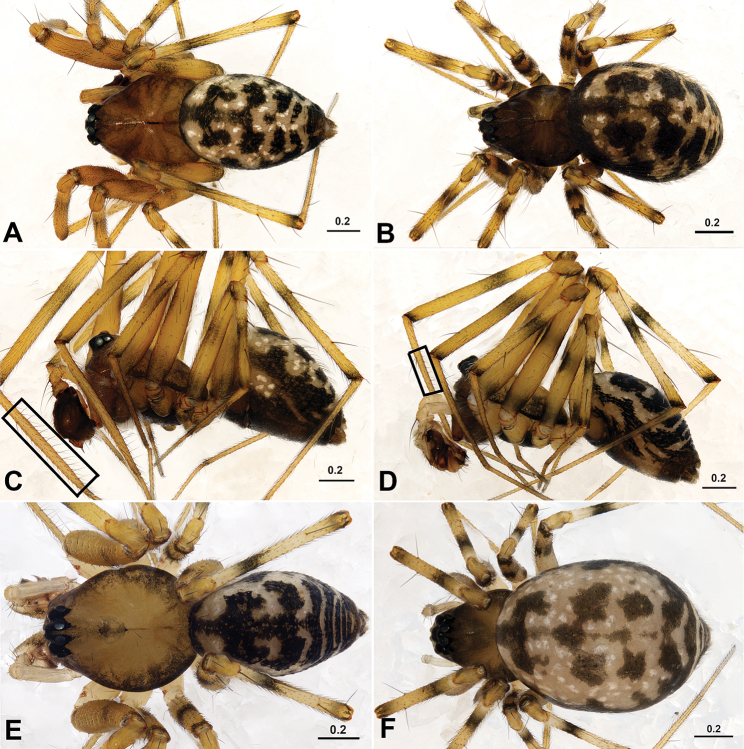
*Acanoides beijingensis* sp. n. (**A–C**) and *Acanoides hengshanensis* (**D–F**). **A** male, dorsal **B** female, dorsal **C** male, lateral, rectangle indicates ventrolateral rows of bristles on Mt I **D** male, lateral, rectangle indicates ventrolateral rows of bristles on Mt I **E** male, dorsal **F** female, dorsal. [Scale bars: mm].

Male total length 2.34–2.73; female total length 2.10–2.42. Carapace yellowish-brown. Male carapace unmodified. AMEs smallest, others subequal; from the dorsal view AER recurved, PER straight, eyes separated by AMEd, ALE and PLE juxtaposed. Chelicerae medium-sized, with strong stridulatory ridges, female fang groove with three promarginal and three retromarginal teeth in *Acanoides beijingensis* sp. n., and two promarginal and two retromarginal teeth in *Acanoides hengshanensis*. Chaetotaxy: Ti I–IV: 2-2-2-2; Mt I–IV: 1-1-1-1; Mt I of males with two rows of ventral bristles, one prolateral, one retrolateral (Fig. 1C, 1D); Tm I about 0.25, Tm IV absent. Both species have a haplotracheate system.

**Figure 2.**
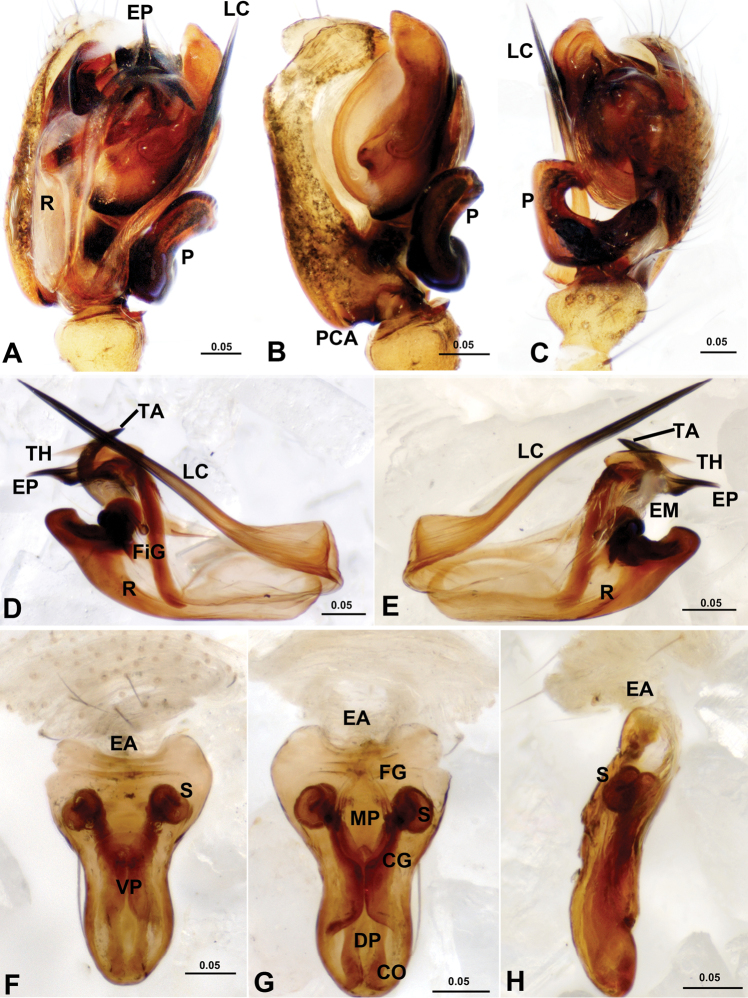
*Acanoides beijingensis* sp. n. **A** male palp, prolateral **B** male palp, prolateral, with embolic division removed **C** male palp, retrolateral **D** embolic division, ventral **E** embolic division, dorsal **F** epigynum, ventral **G** epigynum, dorsal **H** epigynum, lateral. CG copulatory groove; CO copulatory opening; DP dorsal plate; EA extensible area of epigynal basal part; EM embolic membrane; EP embolus proper; FG fertilization groove; FiG Fickert’s gland; LC lamella characteristica; MP median plate; P paracymbium; PCA proximal cymbial apophysis; R radix; S spermathecae; TA terminal apophysis; TH thumb of embolus; VP ventral plate. [Scale bars: mm].

**Figure 3.**
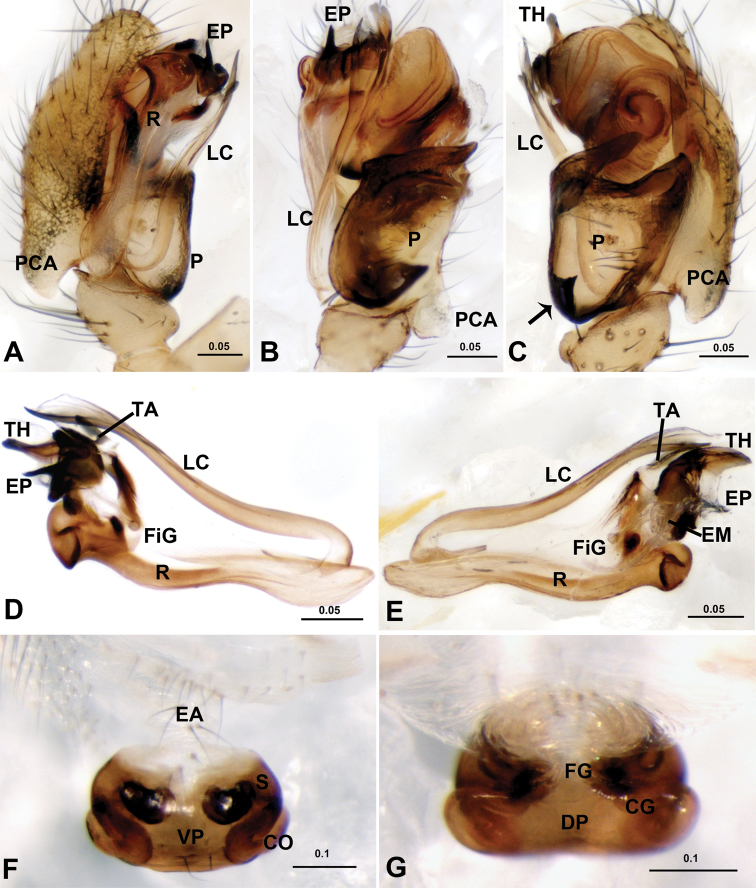
*Acanoides hengshanensis*. **A** male palp, prolateral **B** male palp, ventral **C** male palp, retrolateral, arrow indicates pointed tooth on posterolateral margin **D** embolic division, ventral **E** embolic division, dorsal **F** epigynum, ventral **G** epigynum, dorsal. CG copulatory groove; CO copulatory opening; DP dorsal plate; EA extensible area of epigynal basal part; EM embolic membrane; EP embolus proper; FG fertilization groove; FiG Fickert’s gland; LC lamella characteristica; P paracymbium; PCA proximal cymbial apophysis; R radix; S spermatheca; TA terminal apophysis; TH thumb of embolus; VP ventral plate. [Scale bars: mm].

**Figure 4.**
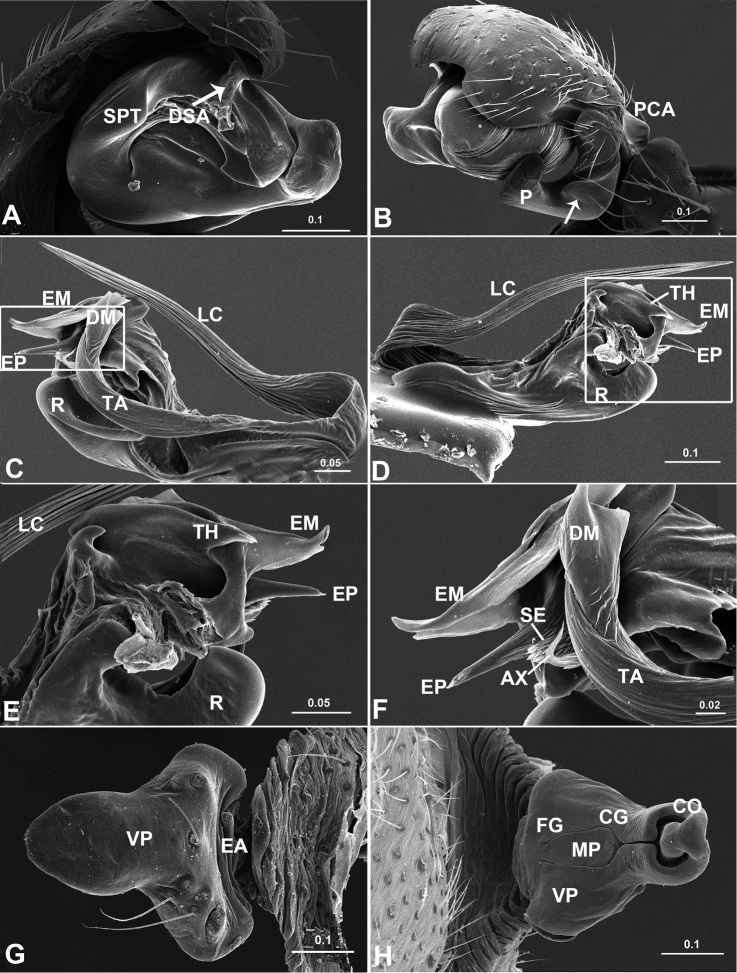
*Acanoides beijingensis* sp. n. **A** palp (embolic division removed), prolateral **B** palp, retrolateral, arrow indicates half rounded lateral tooth on paracymbium **C** embolic division, ventral **D** embolic division, dorsal **E** detail of D **F** detail of C **G** epigynum, ventral **H** epigynum, dorsal. AX apex of embolus; CG copulatory groove; CO copulatory opening; DM distal membrane of terminal apophysis; DSA distal suprategular apophysis; EA extensible area of epigynal basal part; EM embolic membrane; EP embolus proper; FG fertilization groove; LC lamella characteristica; MP median plate; P paracymbium; PCA proximal cymbial apophysis; R radix; S spermatheca; SE serrated area on embolus; SPT suprategulum; TA terminal apophysis; TH thumb of embolus; VP ventral plate. [Scale bars: mm].

**Figure 5.**
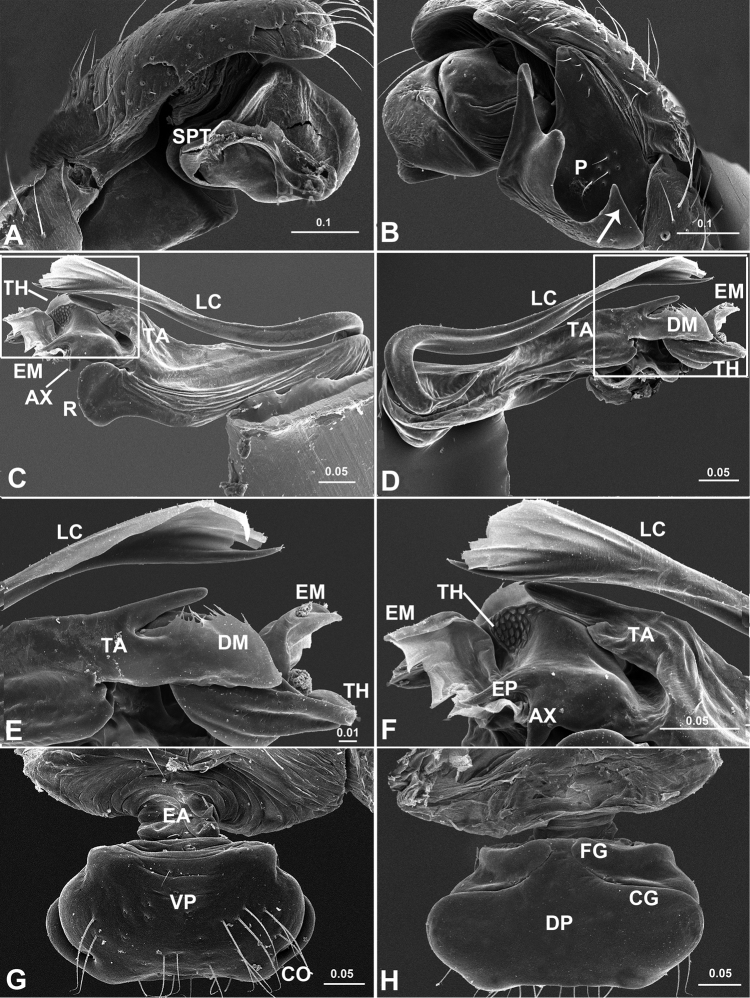
*Acanoides hengshanensis*. **A** palp (embolic division removed), prolateral **B** palp, retrolateral, arrow indicates pointed tooth on posterolateral margin **C** embolic division, ventral **D** embolic division, dorsal **E** detail of D **F** detail of C **G** epigynum, ventral **H** epigynum, dorsal. AX apex of embolus; CG copulatory groove; CO copulatory opening; DM distal membrane of terminal apophysis; EA extensible area of epigynal basal part; EM embolic membrane; EP embolus proper; FG fertilization groove; LC lamella characteristica; P paracymbium; PCA proximal cymbial apophysis; R radix; S spermatheca; SPT suprategulum; TA terminal apophysis; TH thumb of embolus; VP ventral plate. [Scale bars: mm].

*Male palp* (Figs 2A–E, 3A–E, 4A–B, 5A–B). Cymbium with proximal apophysis. Paracymbium medium to large-sized, with one tooth on lateral margin. Distal suprategular apophysis not modified as pit hook, or absent. Embolic division: radix long and narrow, Fickert’s gland located in the membranous area connecting radix and embolus; embolus wide and strongly sclerotized with serrated area, embolus proper sharp with a thumb and an apex at each side; lamella characteristica unbranched, long and narrow with sharp sclerotized apex, almost parallel to radix; terminal apophysis with distal membrane.

*Epigynum* (Figs 2F–H, 3F–G, 4G–H, 5G–H). Protruding, with deeply wrinkled basal part, extensible and ventrally folded in constricted state. Epigynum well sclerotized, epigynal cavity present (in *Acanoides beijingensis* sp. n.) or absent (in *Acanoides hengshanensis*), both scape and stretcher absent.

##### Etymology.

The genus name, *Acanoides*, is a combination of the first four letters of “*Acanthoneta*” and the last five letters of “Wubanoides”. “-oides” itself in Latin means “similar to”, masculine in gender.

##### Phylogenetics.

Due to limitations of the current dataset the monophyly of *Acanoides* could not be tested explicitly in our phylogenetic analyses, however it is supported by the following four putative synapomorphies: sharp embolus proper, slender and unbranched lamella characteristica, outside radix located Fickert’s gland and ventrally folded extensible epigynal basal part.

##### Distribution.

China (Beijing, Hunan, Hebei) (Fig. 7).

##### Remarks.

**Figure 6.**
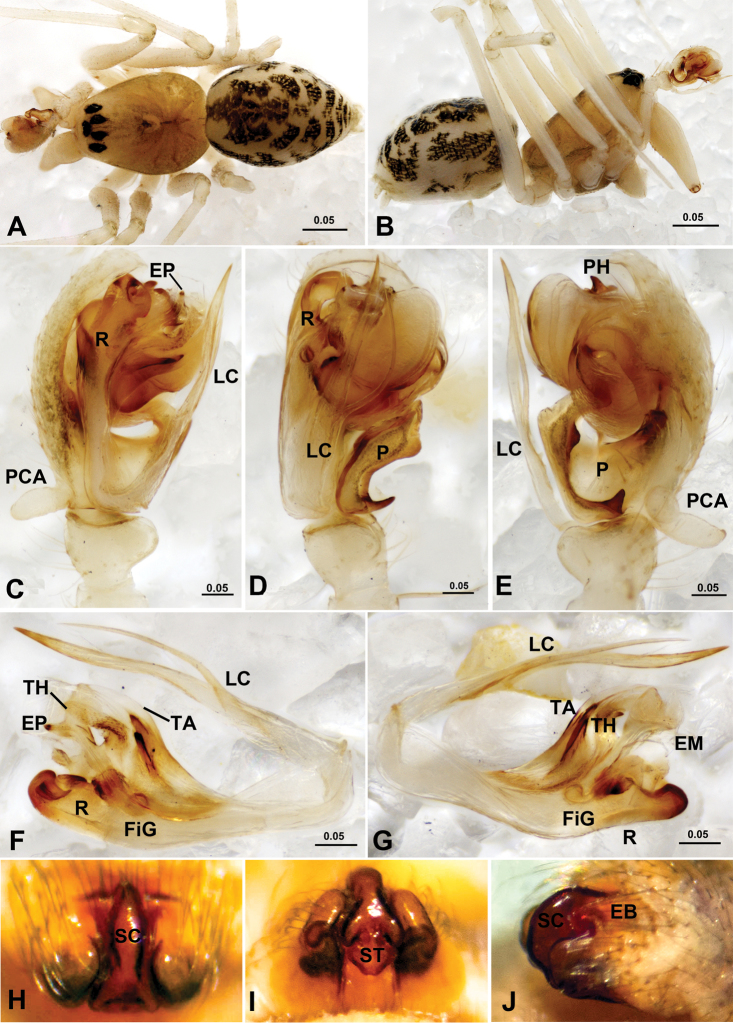
*Acanthoneta dokutchaevi* (**A–G**) and *Acanthoneta aggressa* (**H–J**). **A** male, dorsal **B** male, lateral **C** male palp, prolateral **D** male palp, ventral **E** male palp, retrolateral **F** embolic division, ventral **G** embolic division, dorsal **H** epigynum, ventral **I** epigynum, posterior **J** epigynum, lateral (**H–J** photos provided by Don Buckle). EB epigynal basal part; EM embolic membrane; EP embolus proper; FiG Fickert’s gland; LC lamella characteristica; P paracymbium; PCA proximal cymbial apophysis; PH pit hook; R radix; SC scape; ST stretcher; TA terminal apophysis; TH thumb of embolus. [Scale bars: mm].

The males of *Acanoides* gen. n. have the palp of a “micronetine” type: presence of the Fickert’s gland, the boat-shaped radix, the trunk-like embolus with a pointed proper and a thumb, as well as the well developed terminal apophysis and lamella characteristica (Saaristo and Tanasevitch 1996). However, these sclerites in *Acanoides* (Fig. 2D) have some features different from the normal “micronetine” type (Fig. 6F, Saaristo and Tanasevitch 1996): Fickert’s gland is not embedded within the radix, but located in the membranous area connecting the radix and the embolus; and the embolus has a wide, strongly sclerotized body, with a longer and sharper embolus proper, whereas in most “micronetines” the embolus is usually trunk-like with a pointed embolus proper. The male palp of both *Acanoides* and *Acanthoneta*, have a long and slender lamella characteristica parallel to the long radix, but with an additional long and thin branch in *Acanthoneta* (Fig. 6F), unbranched in *Acanoides* (Figs 2D, 3D). The epigynum of *Acanthoneta* is in a normal “micronetine” type, with a sigmoid scape surrounded by an epigynal cavity (Fig. 6H), but with an extensible basal part in *Acanoides*.

**Figure 7.**
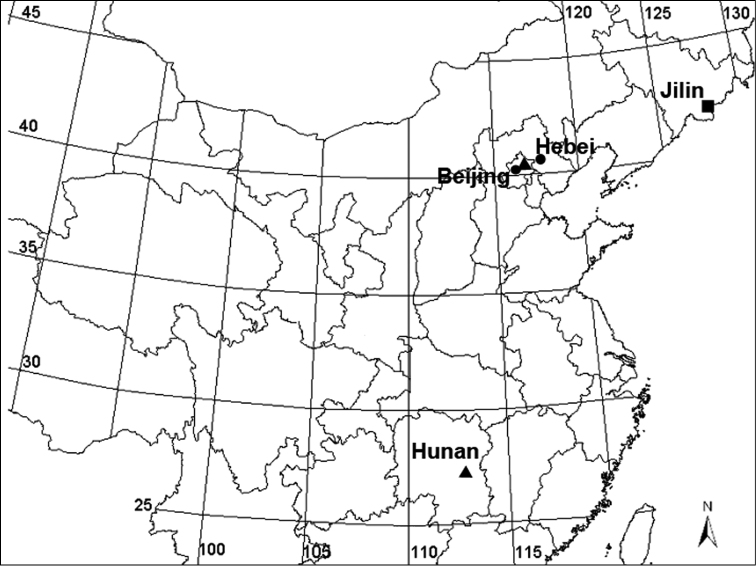
Collecting localities of *Acanoides* species and *Acanthoneta aggressa*, *Acanoides beijingensis* sp. n. (Beijing, Hebei); *Acanoides hengshanensis* (Hunan, Beijing); *Acanthoneta dokutchaevi* (Jilin).

The result of phylogenetic analysis based on molecular data indicates that Ipainae is not a monophyletic group as the movable epigynum independently evolved in *Acanoides* and *Solenysa* (Appendix - Fig. S1). This is also supported by the tracheal characters: haplotracheate type in *Acanoides*, but intermediate type in *Solenysa*, with the median pair extending into the prosoma (Tu and Hormiga 2011). We infer that the extensible basal part of the epigynum may have also evolved convergently with that in other ipaines. In *Acanoides* it differs by being ventrally folded, while it forms a solenoid in *Solenysa* (Tu & Hormiga, 2011), and folds inwards in other ipaines, e.g. *Ipa* (Saaristo 2007: fig. 29), *Wubanoides* and *Epibellowia* (Tanasevitch 1996: figs 7–9). Furthermore, the male palp of typical ipaines has filiform embolus proper (Saaristo 2007: fig. 7; Tanasevitch 1996: figs 1, 4) much longer than that of *Acanoides* (Fig. 2D).

#### Acanoides beijingensis sp. n.

http://zoobank.org/CE596A12-9C21-4B8F-97FC-F31CBC61CD7E

http://species-id.net/wiki/Acanoides_beijingensis

Figs 1A–C , 2 , 4

##### Type-locality.

China, Beijing: Mt. Yangtaishan, 39°20.15'N, 115°34.52'E, alt. ca 320m, 15 Oct. 2007, L. Tu leg.

##### Type-specimens.

Holotype, ♂ (CNU), China, Beijing, Mt. Yangtaishan, 39°20.15'N, 115°34.52'E, alt. ca 320 m, 15 Oct. 2007, L. Tu leg. Paratypes, 2 ♂♂ and 3 ♀♀ (CNU), same data as holotype.

##### Additional material examined.

1 ♂ and 2 ♀♀ (CNU), China, Hebei Province, Mt. Wulingshan, 40°33.61'N, 117°29.69'E, alt. ca 1100 m, 12 Aug. 2009, L. Tu leg.

##### Diagnosis.

The male of *Acanoides beijingensis* sp. n. can be distinguished from *Acanoides hengshanensis* by the spine-shaped lamella characteristica (Figs 2D, 4C), ribbon-like in the latter (Figs 3D, 5C); by the hook-shaped terminal apophysis (Fig. 4C), straight in the latter (Fig. 5D); and by the presence of a distal suprategular apophysis (Fig. 4A), absent in the latter. The female is distinct by having the epigynum two times longer than wide (Fig. 2F), shorter than wide in *Acanoides hengshanensis* (Fig. 3F); and by the presence of a remnant epigynal cavity (Fig. 2G), totally absent in *Acanoides hengshanensis* (Fig. 3G).

##### Description.

Male holotype (Fig. 1A, C): Total length 2.69. Carapace 1.22 long, 1.01 wide. Abdomen 1.39 long, 0.88 wide. Lengths of legs: I 3.88 (1.05 + 1.18 + 0.99 + 0.66); II 3.02 (1.03 + 0.73 + 0.69 + 0.57); III 2.66 (0.87 + 0.88 + 0.51 + 0.40); IV 3.78 (1.12 + 1.09 + 0.93 + 0.64). Female (Fig. 1B): Total length 2.12. Carapace 0.93 long, 0.78 wide. Abdomen 1.25 long, 0.83 wide. Lengths of legs: I 6.10 (1.68 + 2.04 + 1.43 + 0.95); II 5.43 (1.56 + 1.74 + 1.24 + 0.89); III 4.39 (1.24 + 1.13 + 1.10 + 0.75); IV 5.88 (1.79 + 1.78 + 1.46 + 0.83). Tm I: 0.20. For other somatic features see description of the genus.

*Male palp* (Figs 2A–C, 4A–B). Cymbium with proximal apophysis. Paracymbium narrow, half rounded lateral tooth strongly sclerotized. Distal suprategular apophysis blunt, not modified as pit hook. Embolic division: radix long and narrow; Fickert’s gland located in the membranous area connecting radix and embolus; embolus main body short and wide, strongly sclerotized, with serrated area on ventral surface; embolus proper sharp with pointed thumb and tail-like apex at each side; unbranched lamella characteristica long and slender, with sharp and strongly sclerotized apex; terminal apophysis hook-shaped with distal membrane.

*Epigynum* (Figs 2F–H, 4G–H). Two times longer than wide, wrinkled basal part extensible and ventrally folded in constricted state. Median plate and epigynal cavity present, without scape and stretcher. Copulatory openings opened dorsally.

##### Etymology.

The species name refers to the type locality.

##### Variation.

*Males* (n = 3). Total length 2.61–2.73. Carapace: 1.13–1.27 long, 0.95–1.05 wide. Abdomen 1.34–1.45 long, 0.71–0.99 wide.

*Females* (n = 3). Total length 2.10–2.23. Carapace: 0.90–0.96 long, 0.74–0.78 wide. Abdomen: 1.10–1.38 long, 0.79–0.88 wide.

##### Distribution.

China (Beijing, Hebei) (Fig. 7).

##### Remarks.

Although *Acanoides beijingensis* sp. n. looks quite different from *Acanoides hengshanensis* in the shape of the male paracymbium and in terms of female epigynal morphology, the strongly sclerotized embolus main body and the sharp embolus proper, the location of Fickert’s gland, the presence of a ventrally folded extensive area of the epigynal basal part and the absence of a scape and stretcher, shared by the two species suggest they are closely related. A close relationship between the two species is additionally supported by the phylogenetic analysis (Appendix - Fig. S1).

#### Acanoides hengshanensis

(Chen & Yin, 2000) comb. n.

http://species-id.net/wiki/Acanoides_hengshanensis

Figs 1D–F , 3 , 5

Lepthyphantes hengshanensis Chen & Yin, 2000: 87, figs 12–16 (♂)
Acanthoneta hengshanensis : Tu et al. 2006: 412, figs 24–27 (♂).

##### Type-specimen.

Holotype of *Lepthyphantes hengshanensis* Chen & Yin, 2000, ♂ (HNU), China, Hunan Province, Mt. Hengshan, 27°18'N, 112°42'E, 1–7 Aug. 1995, C. Yin leg. (examined).

##### Additional material examined.

3 ♂♂ and 4 ♀♀, China, Beijing, Mt. Yangtaishan, Dajue Temple, 40°03.06'N, 116°05.97'E, alt. 50 m, 15 Oct. 2007, L. Tu leg.

##### Diagnosis.

See diagnosis for *Acanoides beijingensis* sp. n.

##### Description.

Male (Fig. 1D–E): Total length 2.39. Carapace 1.02 long, 0.78 wide. Abdomen 1.37 long, 0.78 wide. Lengths of legs: I 5.03 (1.37 + 1.56 + 1.32 + 0.78), II 3.33 (0.98 + 0.98 + 0.83 + 0.54), III 3.47 (0.98 + 1.07 + 0.88 + 0.54), IV 4.63 (1.27 + 1.41 + 1.22 + 0.73). Tm I: 0.24. Female (Fig. 1F): Total length 2.42. Carapace 0.96 long, 0.78 wide. Abdomen 1.80 long, 1.25 wide. Lengths of legs: I 4.21 (1.18+ 1.42 + 0.96 + 0.65), II 3.19 (0.98 + 1.06 + 0.66 + 0.49), III 2.81 (0.84 + 0.85 + 0.68 + 0.44), IV 3.70 (1.08 + 1.19 + 0.89 + 0.54). Tm I: 0.23. For other somatic characters see description of the genus.

*Male palp* (Figs 3A–C; 5A–B). Cymbium with distinct proximal apophysis pointing backwards. Paracymbium wide and U-shaped, with triangular tooth on posterolateral margin. Distal suprategular apophysis absent. Embolic division: radix long and narrow; Fickert’s gland located in the membranous area connecting radix and embolus; embolus main body large and strongly sclerotized with serrated area; embolus proper sharp with large thumb and pointed apex; lamella characteristica long and slender with bifurcated ends, one sharp and sclerotized, one membranous; terminal apophysis straight, with distal membrane.

*Epigynum* (Figs 3F–G, 5G–H). Short and wide, box-shaped, strongly sclerotized; wrinkled basal part extensible and ventrally folded in constricted state. Neither median plate nor epigynal cavity present. Copulatory openings located on ventral surface, slits of epigynal grooves extending laterally, passing from ventral to dorsal surface, then convergent mesally. No scape, no stretcher.

##### Variation.

*Males* (n = 3). Total length 2.34–2.41. Carapace: 1.09–1.12 long, 0.72–0.93 wide. Abdomen 1.14–1.42 long, 0.68–0.83 wide.

*Females* (n = 4). Total length 2.32–2.42. Carapace: 0.87–1.01 long, 0.75–0.81 wide. Abdomen: 1.63–1.82 long, 0.76–1.22 wide.

##### Distribution.

China (Beijing, Hunan) (Fig. 7).

#### Genus Acanthoneta

Eskov & Marusik, 1992 stat. n.

Acanthoneta Eskov & Marusik, 1992: 34. Described as a subgenus of *Poeciloneta*.
Acanthoneta : Saaristo and Tanasevitch 1996: 175. Raised to generic status without any comments or argumentation.

##### Type species.

*Poeciloneta aggressus* (Chamberlin & Ivie, 1943).

##### Composition.

Three species: *Acanthoneta aggressa* Chamberlin & Ivie, 1943 (Nearctic), *Acanthoneta dokutchaevi* Eskov & Marusik, 1993 (Far East Asia) and *Acanthoneta furcata* Emerton, 1913 (Nearctic).

##### Comments.

Originally *Acanthoneta* was described as a subgenus of *Poeciloneta*, including two species: *Poeciloneta (Acanthoneta) aggressa* and *Poeciloneta (Acanthoneta) furcata*. One additional species *Acanthoneta dokutchaevi* was assigned to the subgenus by Eskov and Marusik (1993). Saaristo and Tanasevitch (1996) raised *Acanthoneta* to genus status without any argumentations and hence the new status was not accepted by Platnick (2014). Here we provide a diagnosis for *Acanthoneta* and a comparison with *Poeciloneta*.

##### Diagnosis.

Males of *Acanthoneta* differ from *Poeciloneta* by the long radix almost parallel with the long lamella characteristica (Fig. 6F), in the latter the radix is normal boat-shaped, lamella characteristica large and ribbon-like (Saaristo and Tanasevitch 2000: fig. 11). Females of the two genera differ by the epigynum in *Acanthoneta* having a sigmoid scape surrounded by an epigynal cavity, the lateral wall of which is posteriorly extended (Fig. 6H), whereas in *Poeciloneta* the scape is exposed, enlarged and strongly sclerotized (Saaristo and Tanasevitch 2000: fig. 18).

#### Acanthoneta aggressa

(Chamberlin & Ivie, 1943)

http://species-id.net/wiki/Acanthoneta_aggressa

Fig. 6H–J

Lepthyphantes aggressus Chamberlin & Ivie, 1943: 14, figs 19–20.
Poeciloneta aggressa : Crawford 1988: 19.
Acanthoneta aggressa : Saaristo and Tanasevitch 1996: 175.
Poeciloneta aggressa : Paquin and Dupérré 2003: 147, figs 1623–1625.

##### Material examined.

No material examined, epigynum pictures were provided by Don Buckle (Saskatoon, Canada): 1 ♀, Canada, Alberta, Chinook Lake, under log in spruce or fir woods, 49°40'N, 114°30'W, 25 Jul. 1988, D. J. Buckle leg.

##### Description.

*Epigynum* (Fig. 6H–J). Slightly protruding, without extensible area at basal part. Epigynal cavity, with posteriorly extended lateral wall, surrounding sigmoid folded scape; scape long and narrow, with well developed lateral lobes hosting copulatory openings and distal stretcher.

##### Distribution.

Across North America from Washington State to Québec (Buckle et al. 2001; Paquin and Dupérré 2003).

#### Acanthoneta dokutchaevi

Eskov & Marusik, 1993

http://species-id.net/wiki/Acanthoneta_dokutchaevi

Fig. 6A–G

Poeciloneta (Acanthoneta) aggressa non Chamberlin & Ivie, 1943: Eskov and Marusik 1992: 34–35, figs 11–13 (♂).
Poeciloneta (Acanthoneta) dokutchaevi : Eskov and Marusik 1993: 52, figs 49–51 (♂).

##### Material examined.

1 ♂, China, Jilin Province, Mt. Changbaishan, Ski. 42°01.54'N, 128°04.25'E, alt. ca 1260 m, 31 July 1971.

##### Description.

Male (Fig. 6A–B). Chelicera long, with strong stridulatory ridges. Chaetotaxy: Ti I–IV: 2-2-2-2; Mt I–IV: 1-0-0-1; Tm I about 0.80, Tm IV present. For other somatic characters see description by Eskov and Marusik (1993).

*Male palp* (Fig. 6C–E). Cymbium with proximal apophysis erected. Paracymbium wide, with two pointed teeth on lateral margin. Distal suprategular apophysis modified as pit hook. Embolic division: radix long and narrow; Fickert’s gland located within radix; embolus main body trunk-like with serrated area, pointed embolus proper and well developed thumb; lamella characteristica fork-like branched, long and slender, almost parallel to radix; terminal apophysis with distal membrane and two strongly sclerotized teeth on ventral side.

Female. Unknown.

##### Remarks.

The male of this species is similar to the type species *Acanthoneta aggressa*. It differs only by the shape of the paracymbium. For a detailed comparison see Eskov and Marusik (1993).

##### Distribution.

Far East Asia: Magadan Area (Eskov and Marusik 1993) and China (Fig. 7) (new record).

## Supplementary Material

XML Treatment for Acanoides

XML Treatment for Acanoides beijingensis

XML Treatment for Acanoides hengshanensis

XML Treatment for Acanthoneta

XML Treatment for Acanthoneta aggressa

XML Treatment for Acanthoneta dokutchaevi

## Appendix

**Table S1.** GenBank accession numbers. Data of the taxa labeled with “#” are newly sequenced; the taxa labeled with “*” come from Arnedo et al. 2009.

Family	Genus Species	16s	18s	28s	COI	H3
Araneidae	*Argiope trifasciata*		FJ525386	FJ525368	FJ525316	FJ525335
Cyatholipidae	*Alaranea merina**	AY230942	AY230890	AY231074	AY231022	AY230982
Mysmenidae	*Maymena ambita*	GU456746	GU456765	GU456824	GU456876	GU456921
Nesticidae	*Nesticus cellulanus*	EU746444	AF005447	AF124961	EU746435	
Pimoidae	*Pimoa haden*	GU338640	GU338524	EF128112	EF128155	
Pimoidae	*Pimoa* sp.*	AY230940	AY230893	AY231072	AY231025	AY230985
Synotaxidae	*Synotaxus* sp.	AY230943	AY230894	AY231076	AY231026	AY230986
Theridiidae	*Steatoda bipunctata**	AY230951	AY230926	AY231084	AY231057	AY231014
Theridiidae	*Theridion varians**	AY230976	AY230932	AY231111	AY231063	AY231017
Theridiosomatidae	*Theridiosoma gemmosum*	HM030408	HM030417	HM030428	HM030436	HM030443
Linyphiidae	*Acanoides beijingensis*^#^	KJ027589	KJ027587	KJ027580	KJ027582	KJ027583
	*Acanoides hengshanensis*^#^	KJ027585	KJ027588	KJ027584	KJ027586	KJ027581
	*Acanthoneta* sp.		GU338479	GU338560	GU338678	
	*Agyneta* sp.	GU338621		GU338529		
	*Agyneta ramosa**	FJ838670	FJ838694	FJ838717	FJ838648	FJ838740
	*Anguliphantes karpinskii*		GU338516	GU338566	GU338680	
	*Asperthorax communis*		GU338482	GU338545	GU338684	
	*Asthenargus* sp.		GU338493	GU338561		
	*Australolinyphia remota**	FJ838671	FJ838695	FJ838718	FJ838649	FJ838741
	*Bathyphantes floralis*	GU338604	GU338465	GU338583	GU338659	
	*Bathyphantes gracilis**	FJ838672	FJ838696	FJ838719	FJ838650	FJ838742
	*Bathyphantes gracilis*	GU338630	GU338464	GU338582	GU338689	
	*Bolyphantes alticeps**	AY078660	AY078667	AY078678	AY078691	AY078700
	*Capsulia* sp.		GU338470	GU338586		
	*Centromerus trilobus*	GU338599	GU338468	GU338571	GU338656	
	*Collinsia inerrans*	GU338601	GU338518		GU338645	
	*Collinsia plumose*	GU338638	GU338499	GU338543		
	*Denisiphantes* sp.	GU338619	GU338508	GU338563	GU338669	
	*Dicymbium sinofacetum*	GU338614	GU338487	EF128119	GU338665	
	*Diplocentria bidentata*	GU338629	GU338494	GU338542	GU338688	
	*Diplocephalus cristatus*	GU338637	GU338490		GU338696	
	*Diplostyla concolor**	FJ838673	FJ838697	FJ838720	FJ838651	FJ838743
	*Diplostyla concolor*	GU338639	GU338467	GU338585	GU338697	
	*Doenitzius peniculus*	GU338631	GU338469		GU338690	
	*Doenitzius pruvus*	GU338632	GU338474		GU338691	
	*Drapetisca socialis**	FJ838674	FJ838698	FJ838721	FJ838652	FJ838744
	*Dubiaranea aysenensis*	FJ838675	FJ838699	FJ838722	FJ838653	FJ838745
	*Dubiaranea distincta*	GU338624	GU338459	GU338579	GU338648	
	*Dubiaranea propinquua*	GU338627	GU338460	GU338580	GU338675	
	*Dubiaranea similis*		GU338521	GU338581	GU338681	
	*Erigone edentate*		GU338486	GU338540	GU338686	
	*Erigone prominens*		GU338498	GU338539	GU338679	
	*Floronia bucculenta**	FJ838676	FJ838700	FJ838723	FJ838654	FJ838746
	*Frontinella communis*	GU338628	GU338517	GU338573		
	*Frontinella communis**	FJ838677	FJ838701	FJ838724	FJ838655	FJ838747
	*Fusciphantes hibanus*		GU338512	GU338570	GU338683	
	*Gnathonarium dentatum*	GU338593	GU338477	EF128120	GU338651	
	*Gnathonarium taczanowskii*	GU338620	GU338480	GU338547	GU338670	
	*Gonatium japonicum*	GU338613	GU338492			
	*Gonatium rubellum**	FJ838679	FJ838703	FJ838726	FJ838656	FJ838749
	*Gongylidiellum vivum**	FJ838678	FJ838702	FJ838725		FJ838748
	*Grammonota* sp.		GU338491		GU338685	
	*Haplinis diloris**	FJ838680	FJ838704	FJ838727	FJ838657	FJ838750
	*Helophora insignis**	FJ838681	FJ838705	FJ838728	FJ838658	FJ838751
	*Himalaphantes azumiensis*		GU338522	GU338564	GU338677	
	*Hylyphantes graminicola*	GU338595	GU338478	GU338550	GU338653	
	*Hylyphantes* sp.	GU338618	GU338481	GU338549	GU338668	
	*Labulla thoracica**	AY078662	AY078674	AY078680	AY078694	AY078707
	*Laetesia* sp.*	FJ838682	FJ838706	FJ838729	FJ838659	FJ838752
	*Lepthyphantes minutus**	AY078663	AY078673	AY078681	AY078689	AY078705
	*Lepthyphantes leprosus*		GU338488	GU338565	GU338682	
	*Lepthyphantes* sp.	GU338610	GU338509	GU338562	GU338664	
	*Linyphia triangularis**	AY078664	AY078668	AY078682	AY078693	AY078702
	*Linyphia* sp.	GU338597	GU338461	GU338572	GU338654	
	*Macrargus alpinus*		GU338505	GU338559		
	*Agyneta nigra*	GU338608	GU338504	GU338577	GU338662	
	*Agyneta rurestris**	FJ838683	FJ838707	FJ838730	FJ838660	FJ838753
	*Microlinyphia dana**	AY078665	AY078677	AY078683	AY078690	
	*Microneta* sp.	GU338609	GU338472	GU338538	GU338663	
	*Microneta viaria**	FJ838684	FJ838708	FJ838731	FJ838661	FJ838754
	*Microneta viaria*	GU338598	GU338502	GU338537	GU338655	
	*Moebelia rectangular*	GU338591	GU338485	GU338557		
	*Mughiphantes nigromaculatus*	GU338600	GU338510	GU338527	GU338644	
	*Nematogmus sanguinolentus*	GU338635	GU338489	GU338544	GU338694	
	*Neomaso patagonicus*	GU338626	GU338473	GU338578	GU338674	
	*Neriene japonica*	GU338633	GU338462	GU338575	GU338692	
	*Neriene radiata**	AY078710	AY078670	AY078684	AY078696	AY078709
	*Neriene radiate*	GU338623	GU338463	GU338574	GU338672	
	*Neriene variabilis**	AY078711	AY078669	AY078685	AY078699	AY078706
	*Nesioneta ellipsoidalis*		GU338519	GU338532	GU338687	
	*Nippononeta kantonis*	GU338634	GU338471	GU338530	GU338693	
	*Nippononeta* sp.	GU338602	GU338520	GU338531	GU338657	
	*Notholepthyphantes australis**	FJ838685	FJ838709	FJ838732	FJ838662	FJ838755
	*Novafroneta vulgaris**	FJ838686	FJ838710	FJ838733	FJ838663	FJ838756
	*Oedothorax apicatus**	FJ838687	FJ838711		FJ838664	FJ838757
	*Orsonwelles* malus*	AY078737	AY078676	AY078795	AY078697	AY078708
	*Orsonwelles* polites*	AY078725	AY078671	AY078786	AY078755	AY078701
	*Ostearius melanopygius**	FJ838688	FJ838712	FJ838735		FJ838758
	*Paikiniana* sp.	GU338617	GU338495	GU338555	GU338647	
	*Parameioneta bilobata*	GU338605	GU338503	GU338533	GU338660	
	*Parasisis* sp.	GU338592	GU338500	GU338534	GU338650	
	*Pityohyphantes costatus**	AY078666	AY078675		AY078695	
	*Pocobletus* sp.*	FJ838689	FJ838713	FJ838736	FJ838665	FJ838759
	*Porrhomma* sp.	GU338607	GU338466	GU338584	GU338661	
	*Prosoponoides sinensis*	GU338606		GU338576	GU338649	
	*Pseudafroneta incerta**	FJ838690	FJ838714	FJ838737	FJ838666	FJ838760
	*Ryojius* sp.	GU338611		GU338536		
	*Sisicottus montanus*	GU338625	GU338497	GU338541	GU338673	
	*Solenysa* sp.	GU338616	GU338506	GU338528	GU338667	
	*Sphecozone bicolor*	GU338622	GU338496	GU338553	GU338671	
	*Stemonyphantes lineatus**	FJ838691	FJ838715	FJ838738	FJ838667	FJ838761
	*Stemonyphantes sibiricus**	FJ838692			FJ838668	FJ838762
	*Syedra oii*	GU338615	GU338513	GU338569	GU338666	
	*Tapinopa guttata*		GU338511	GU338558	GU338676	
	*Tenuiphantes ancatus*		GU338515	GU338567		
	*Tenuiphantes* sp.	GU338612	GU338514	GU338568	GU338646	
	*Tenuiphantes tenuis**	FJ838693	FJ838716	FJ838739	FJ838669	FJ838763
	*Ummeliata feminea*	GU338594	GU338475	GU338551	GU338652	
	*Ummeliata insecticeps*		GU338476	GU338552		
	*Walckenaeria clavicornis*	GU338596	GU338483	GU338554		
	*Walckenaeria keikoae*	GU338636	GU338484	GU338556	GU338695	
